# The Effectiveness of Nurse-Led Telecare Consultations Among Patients Who Have Experienced a Stroke: Systematic Review and Meta-Analysis

**DOI:** 10.2196/74149

**Published:** 2025-11-27

**Authors:** Arkers Kwan Ching Wong, Shi Yi Zhou, Xingjuan Tao, Nok Yi Tsui, Vivian Wai Yan Kwok, Robbie Mian Wang, Jonathan Bayuo

**Affiliations:** 1 School of Nursing Faculty of Health and Social Sciences Hong Kong Polytechnic University Hong Kong China (Hong Kong); 2 School of Nursing Shanghai Jiao Tong University Shanghai, Shanghai China; 3 Queen Elizabeth Hospital Kowloon Central Cluster Hospital Authority Kowloon China (Hong Kong)

**Keywords:** quality of life, remote consultation, stroke survivors, systematic review, telemedicine, nurse led

## Abstract

**Background:**

Nurse-led telecare consultations have emerged as a promising approach for the long-term management of stroke survivors, particularly in the context of COVID-19 pandemic–related disruptions. While several studies have explored its use, the effectiveness of nurse-led telecare consultations in post–acute stroke care remains unclear.

**Objective:**

This study aimed to evaluate the effectiveness of nurse-led telecare consultation for poststroke management among stroke survivors who were discharged from the hospital and lived in the community.

**Methods:**

A systematic search was conducted across 6 databases—CINAHL, MEDLINE, PsycINFO, PubMed, Embase, and CENTRAL—for randomized controlled trials published from inception to February 2025. Included studies examined nurse-led telecare consultations compared to usual care among stroke survivors living in the community. Studies involving individuals who were hospitalized or institutionalized were excluded, along with reviews, abstracts without full texts, non-English or non-Chinese articles, and studies not meeting the criteria for randomized controlled trials. Primary and secondary outcomes included blood pressure (BP), psychological burden, quality of life, medication adherence, health care service use, stroke recurrence, survivor functioning, and coping. Continuous outcomes were analyzed using mean differences (MDs) or standardized MDs with 95% CIs under a random-effects model and dichotomous outcomes using odds ratios with 95% CIs via the Mantel-Haenszel method. Heterogeneity was assessed using the chi-square test and *I*^2^ statistics.

**Results:**

In total, 9 studies involving 2524 participants were included. Ischemic stroke was the most common type (n=1568, 62.13%) of stroke. Meta-analysis showed that nurse-led telecare significantly increased the likelihood of achieving target BP (odds ratio 2.33, 95% CI: 1.83-2.98; *P*<.001). For continuous outcomes, pooled analyses showed nonsignificant but directionally favorable reductions in systolic BP (MD –4.83, 95% CI –12.51 to 2.85; *I*^2^ =92%), diastolic BP (MD –6.41, 95% CI –13.76 to 0.93; *I*^2^=97%), and low-density lipoprotein cholesterol (MD 0.01, 95% CI –0.08 to 0.09; *I*^2^=97%). Heterogeneity was substantial for several key outcomes (*I*^2^>90% for systolic BP and diastolic BP). Some outcomes, such as medication adherence and stroke recurrence, were reported by only 1 (11.11%) study. Additional benefits were observed in coping ability and reduced hospital readmissions, but findings for psychological well-being and quality of life were mixed.

**Conclusions:**

Nurse-led telecare consultations may support better BP management and coping and reduce hospital readmissions among community-dwelling stroke survivors. However, the pooled effects for continuous outcomes were inconclusive, and heterogeneity remained high. Therefore, these findings should be interpreted with caution, and further high-quality trials with standardized outcome measures and longer-term follow-up are warranted to confirm effectiveness.

**Trial Registration:**

PROSPERO CRD42023492692; https://www.crd.york.ac.uk/PROSPERO/view/CRD42023492692

## Introduction

Recurrent strokes are becoming increasingly prevalent. More than 12.2 million individuals worldwide experienced a stroke in 2021, with 1 in every 4 individuals experiencing a recurrent stroke within a year [1]. People experiencing recurrent strokes are more likely to have a poorer functional prognosis and complications, such as infections, seizures, and thromboembolism, than those with index strokes, which increases disability and mortality as well as adds extra financial burdens on stroke survivors and society [2,3]. Meanwhile, stroke survivors and their carers experience challenges transitioning to home-based care after a stroke, which is exacerbated by the sudden nature of stroke and the multiple care settings that stroke survivors use after hospital discharge [4]. To prevent recurrent stroke, a structured, professional-led transitional program should be customized to stroke survivors immediately after their discharge from the acute stroke unit [5].

Nurse-led post–acute stroke consultation is a common practice worldwide to serve and follow up on individuals who have survived a stroke and have just been discharged from the hospital [6]. An advanced practice nurse (APN) in the clinic monitors and supports the health conditions of those who have survived an acute stroke and encourages them to follow a healthy lifestyle. As part of each consultation, the APN, an expert in stroke care, conducts a comprehensive health assessment, establishes realistic goals with stroke survivors, and proposes ways to help them maintain a healthy lifestyle at home. To satisfy the holistic needs of stroke survivors, APNs may also seek the support of other health care professionals, such as dietitians and physiotherapists, if necessary. Furthermore, they provide their family members with knowledge and caregiving skills to cope with the numerous challenges their loved ones face, thereby reducing the physical and psychosocial burden associated with disease management.

Thus far, nurse-led post–acute stroke clinics have been shown to be effective in disease management and in improving the prognosis of stroke survivors. Studies have shown that poststroke clinics led by APNs can improve follow-up care; identify stroke survivors’ abnormal symptoms; refer them to the appropriate units promptly; minimize complications caused by multiple chronic comorbidities; and improve blood pressure (BP), cholesterol, and psychological well-being [4,7,8]. Middleton et al [9] found that the nurse-initiated poststroke interdisciplinary treatments for hyperglycemia and swallowing dysfunction might provide long-term and sustained benefit. Moreover, several studies have shown that nurse practitioner–led structured clinics can reduce unplanned 30-day readmissions for individuals who have experienced a stroke [4,10-12].

Despite these benefits, the COVID-19 pandemic has magnified the problems of clinics in providing accessible and continuous care to individuals after an episode of acute stroke, especially those with hemiplegia or hemiparesis after the index stroke. Physical disabilities, travel expenses, undesired scheduled visits, and long distances to the clinics are major barriers for stroke survivors to attend scheduled on-site follow-ups [13,14]. In addition, the increased awareness of nosocomial infection in the hospital among the public after the COVID-19 pandemic has further limited stroke survivors’ access to receive rehabilitative care from the APNs. In order to make existing poststroke management strategies more effective and sustainable, telecare consultations may be an alternative to improve the outcomes of individuals who have experienced acute stroke beyond the COVID-19 pandemic.

A telecare consultation refers to an online meeting between health care professionals and individuals using audio-video communication tools, such as Zoom and Microsoft Teams [15]. The use of telecare consultation replaces long-distance and face-to-face contacts between APNs and their clients and improves the effectiveness and adherence of disease management through virtual communication and collaboration [16]. In addition to the potential benefits telecare offers in maintaining the quality of care for individuals, studies have identified its cost-effectiveness in the long run [17,18].

A systematic review has suggested that stroke survivors are motivated to use telecare services due to their convenience and flexibility. However, barriers, such as technical issues and unclear use guidelines, still hinder optimal implementation [19]. Several systematic reviews have examined the effectiveness of nurse-led telecare consultations in managing various chronic conditions, yielding mixed results. For example, Kwok et al [20] found no significant difference in health care use between telecare and usual care among patients with cancer undergoing systemic or radiation therapy. In contrast, Kappes et al [21] reported that nurse-led telecare was effective in individuals with hypertension, leading to improvements in BP control, self-efficacy, and cholesterol levels. Compared to these populations, stroke survivors require long-term, multidisciplinary follow-up for rehabilitation and secondary prevention. Telecare consultation may reduce their exposure to risks, such as falls or infections during hospital visits, and help alleviate caregiver burden. Despite its potential, no systematic review has specifically examined the effectiveness of nurse-led telecare consultations for poststroke management among community-dwelling stroke survivors. This review aims to evaluate the impact of nurse-led telecare consultations on a range of poststroke outcomes, including BP, psychological burden, quality of life (QoL), medication adherence, health care service use, stroke recurrence rate, survivor functioning, and coping. By synthesizing current evidence, this review seeks to inform clinical practice and policy decisions regarding the feasibility and value of integrating telecare into routine poststroke care.

## Methods

### Overview

This systematic review and meta-analysis were conducted following the PRISMA (Preferred Reporting Items for Systematic Reviews and Meta-Analyses) 2020 statement [22]. This study was registered in PROSPERO (CRD42023492692).

### Literature Search

Studies were identified by searching articles in 6 databases, including CINAHL, MEDLINE, PsycINFO, PubMed, Embase, and CENTRAL, published from inception to February 2025. A comprehensive list of key search terms included “telerehabilitation,” “post-acute stroke,” “stroke recurrence rate,” “recurrent stroke,” “nurse-led telecare,” “telemedicine,” “telestroke,” “face-to-face nurse-led consultation,” “stroke rehabilitation,” and “randomized controlled trial.” The search strategies of all databases are presented in Multimedia Appendix 1. Identified studies were reviewed concerning the primary and secondary outcomes of stroke survivors after receiving nurse-led consultations in face-to-face and telecare modes. Gray literature, such as unpublished conference papers and government reports, was also searched.

### Eligibility Criteria

Eligibility criteria were developed using the population, intervention, comparison, outcomes, and study design framework. Studies were included if they examined nurse-led telecare consultations for poststroke care compared to usual care among community-dwelling stroke survivors. Randomized controlled trials (RCTs) published in English or Chinese were eligible. Multimedia Appendix 2 summarizes the detailed inclusion and exclusion criteria based on the population, intervention, comparison, outcomes, and study design framework.

### Inclusion and Exclusion Criteria

The inclusion and exclusion criteria for this study are presented in Textbox 1.

**Inclusion and exclusion criteria.**

**Inclusion criteria**

- Participants: studies involving individuals aged 18 years or older who had experienced a stroke and were living independently in the community outside health care facilities were included.
- Interventions: nurse-led telecare consultation was used as the intervention delivery channel in 1 arm of the intervention. The channel could include telephone calls, mobile health apps, videoconferencing, SMS text message, and social media. Nurses should have had at least 50% involvement in the program in terms of frequency or duration of the provision of care.
- Comparisons: studies were included if they examined nurse-led telecare consultations for poststroke care compared to usual care among community-dwelling stroke survivors.
- Outcomes: outcomes included blood pressure, psychological burden, quality of life, medication adherence, health care service use, stroke recurrence rate, survivor functioning, and coping.
- Study design: studies were included if they were randomized controlled trials.

**Exclusion criteria**

- Participants: studies involving patients who were hospitalized or living in assisted residential care facilities, for example, nursing homes or homes for older individuals, were excluded.
- Reviews: scoping reviews, narrative reviews, conference abstracts, articles without published abstracts, and articles without available full texts were excluded. Moreover, articles were excluded if their language was neither English nor Chinese.

### Study Selection

The literature review process is presented in the PRISMA flowchart in the Results section. The search results were retrieved and imported into EndNote (version X9; Clarivate) bibliographic software for screening, and duplicates were removed. The screening process involved 2 stages. First, 2 reviewers (AKCW and SYZ) independently scanned the titles and abstracts according to the selection criteria. Next, full-text articles were identified and assessed for eligibility. Any disagreements were resolved by discussion with a third reviewer (XT).

### Data Extraction

The extracted study characteristics were as follows:

1. Background information of the included study (author, year of publication, study location, and study population)
2. Participants (sample size, average age, gender, stroke severity, and ethnicity)
3. Characteristics of the invention (health care provider, intervention type, delivery medium, study duration, contents of intervention, intervention group, and control group)
4. Comparison (compared between participants with or without exposure to the interventions)
5. Outcomes (time point, outcome variables and measures, and results)

### Quality Assessment

The Cochrane Risk of Bias 2 tool was used to identify the potential risk of bias in the included studies in five domains: (1) bias arising from the randomization process, (2) bias due to deviations from intended interventions, (3) bias due to missing outcome data, (4) bias in measurement of the outcome, and (5) bias in selection of the reported result. The level of bias in each domain and the overall risk of a study were judged as “low risk of bias,” “some concerns,” or “high risk of bias” [23,24].

We assessed the certainty of evidence for each prespecified outcome using the Grading of Recommendations Assessment, Development, and Evaluation (GRADE) approach, considering risk of bias, inconsistency, indirectness, imprecision, and publication bias. Two reviewers (AKCW and SYZ) independently rated the certainty as high, moderate, low, or very low; discrepancies were resolved by discussion with a third reviewer (XT).

### Statistical Analysis

Under the random-effects model (change-from-baseline data were used for continuous outcomes, with SDs derived or imputed where necessary), a mean difference (MD) or a standardized MD with a 95% CI was calculated for continuous outcome data, while odds ratios (ORs) and the 95% CIs were computed using Mantel-Haenszel methods for dichotomous variables. The heterogeneity and significance of the results among studies were assessed using a standard chi-square test. The *I*^2^ value from 30% to 50% was considered moderate heterogeneity, whereas the *I*^2^ value from 50% to 90% represented substantial levels of heterogeneity [23]. Due to the limited number of studies per outcome and substantial variability in intervention characteristics and reporting formats, subgroup analysis, sensitivity analysis, or meta-regression could not be meaningfully performed to investigate the sources of heterogeneity. Publication bias was checked using the visualization of the funnel plot [23].

## Results

### Screening Process

**Figure 1:**
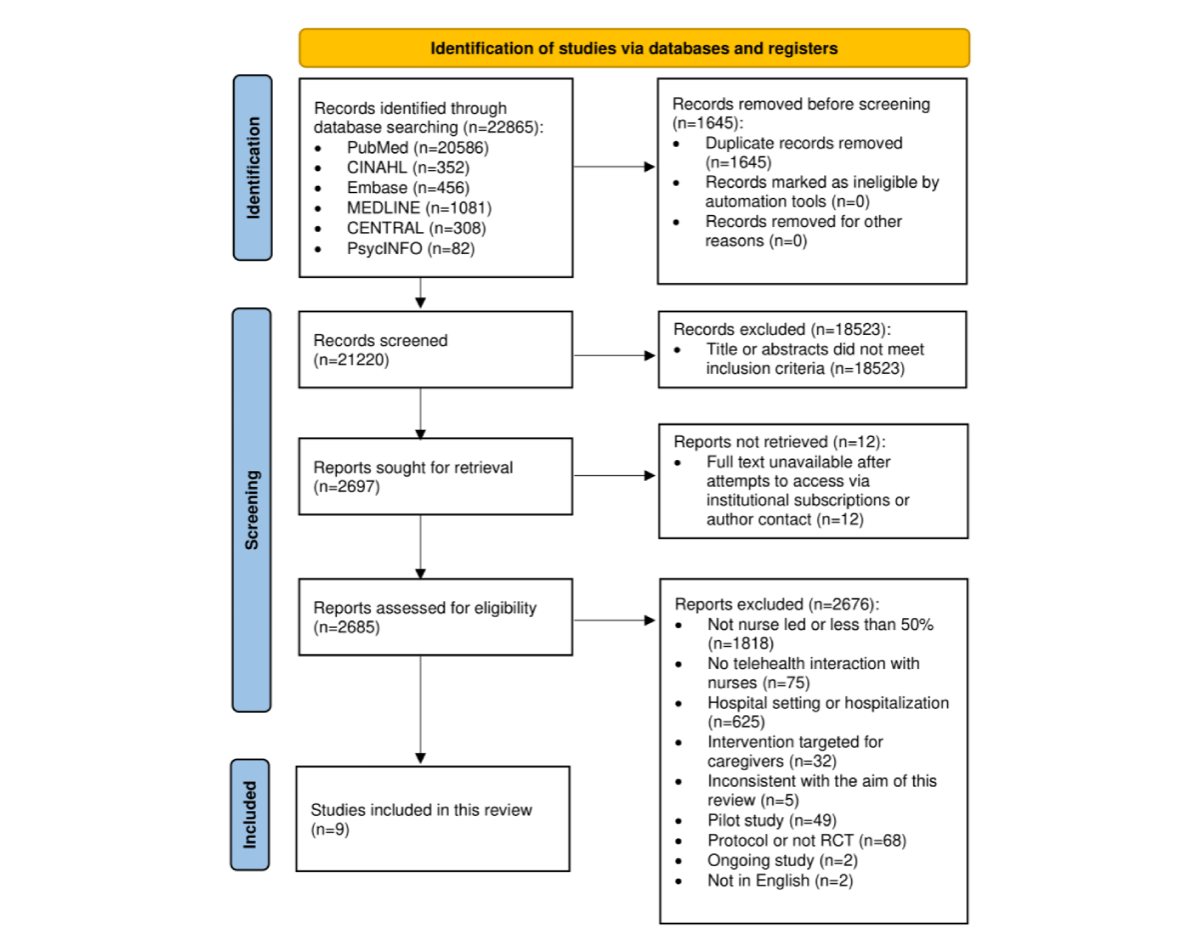
Flow diagram of the identification and selection process according to the PRISMA (Preferred Reporting Items for Systematic Reviews and Meta-Analyses) statement. RCT: randomized controlled trial.

The screening process is reported in Figure 1. A total of 9 (0.34%) studies met the inclusion criteria after a full-text screening of 2685 articles. The characteristics of all included studies are presented in Multimedia Appendix 3 [25-33].

### Study Characteristics

A total of 2524 stroke survivors were included in this review. Of these, 1230 (48.73%) stroke survivors received nurse-led telecare consultation, and 1294 (51.27%) stroke survivors received usual care. Of the 9 included studies, 1 (11.11%) was a 3-armed RCT [25], and the remaining 8 (88.89%) were 2-armed studies. Among the 2524 stroke survivors included, ischemic stroke was the most common type of stroke (n=1568, 62.13%), followed by transient ischemic attack (n=783, 31.04%), hemorrhagic stroke (n=157, 6.22%), and nonspecific stroke (n=16, 0.61%).

### Intervention Characteristics

Of the 9 studies, most (n=5, 55.56%) had interventions delivered by experienced and trained stroke registered nurses, while the other 4 (44.44%) studies were implemented by nurse practitioners (n=2, 22.22%) or clinical nurse specialists (n=2, 22.22%). In 1 (11.11%) study, the intervention was carried out by the rehabilitation team, but the nurses provided more than 50% of the contribution. The duration of the study ranged from 1 to 36 months. Individuals were followed up after discharge for 12 months in most (n=4, 44.44%) studies, while follow-up durations were 6 months (n=2, 22.22%), 3 months (n=1, 11.11%), 1 month (n=1, 11.11%), and 36 months (n=1, 11.11%) in other studies.

Of the 9 studies, most (n=6, 66.67%) used telephone as a service delivery channel [7,26-30], with 2 (22.22%) of them combining remote monitoring [7,26,28]. A total of 3 (33.33%) studies used telerehabilitation [25,30,31], with 1 (11.11%) incorporating telephone counseling and 1 (11.11%) incorporating web-based counseling [30,31]. For studies involving telemonitoring, stroke survivors in the intervention group were asked to attend an outpatient clinic to monitor their BP and low-density lipoprotein cholesterol (LDL-C) and adjust medications based on the recommendations of physicians. Some intervention groups received additional education sessions or brochures before discharge to increase participation and the effectiveness of the intervention. In the study by Boter and the HESTIA study group [27], a checklist on stroke self-management was distributed to stroke survivors. Referral systems were found in some (n=4, 44.44%) studies to facilitate the work of nurses.

The control group received usual care, which mainly consisted of predischarge health education, a health booklet, outpatient on-site follow-up, or regular visits from the researchers.

### Nurse Follow-Ups

Telephone-based counseling by nurses was implemented by 6 (66.67%) of the 9 included studies. The frequency of the intervention was once a month in most (n=4, 44.44%) studies and bimonthly (n=2, 22.22%) or as needed by the stroke survivors (n=2, 22.22%). Contents of the telephone counseling included (1) disease self-management (eg, lifestyle changes, medication adherence, and home BP monitoring; n=5, 55.56%), (2) assessment of medication efficacy (n=2, 22.22%), (3) identifying unmet needs and providing coping strategies (eg, prevention of pressure ulcers, falls, and urinary tract problems; n=2, 22.22%), and (4) referrals to medical services (n=3, 33.33%).

Stroke survivors’ BP was monitored remotely in 3 (33.33%) of the 9 studies. For example, Kerry et al [32] issued a booklet to the intervention group for them to record their daily BP and report back to nurses via telephone. In other studies, stroke survivors who did not meet target BP values received additional telephone counseling to remind them of the techniques of medication taking and self-care skills.

### Telerehabilitation

A total of 3 (33.33%) of the 9 studies involved telerehabilitation. Kirkness et al [25] conducted a 6-session psychological intervention based on cognitive behavioral therapy via phone calls to reduce depression among stroke survivors. Hosseini et al [30] conducted health education over the phone for stroke survivors and their carers to reduce the incidence of poststroke complications. Meanwhile, some (n=5, 55.56%) studies provided suggestions and information on stroke management on a self-developed website for meeting the needs of stroke survivors and their carers. Forums were also provided on the website where nurse specialists and relevant rehabilitation teams could answer stroke survivors’ and their carers’ questions.

### Quantitative Synthesis

**Figure 2:**
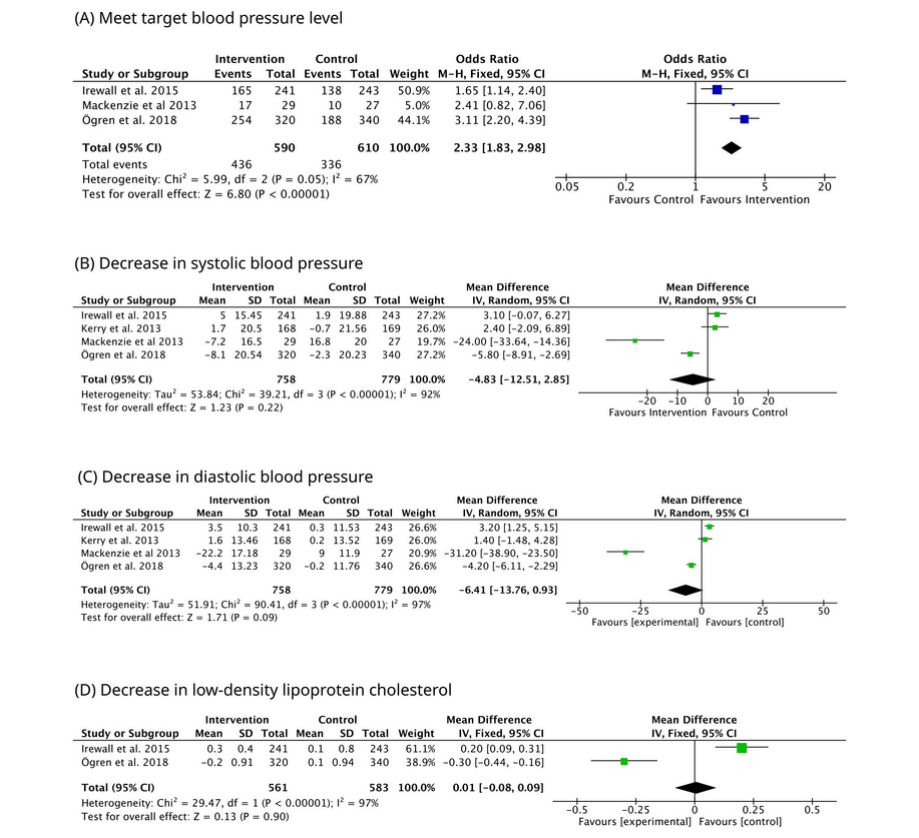
Forest plot on the effectiveness of the nurse-led telecare consultation program on stroke survivors’ blood pressure and low-density lipoprotein cholesterol.

Forest plots for all outcomes are shown in Figure 2 [26,28,32,33].

### BP Status

The proportion of stroke survivors meeting the target BP in the intervention group was statistically significantly greater than in the control group (OR 2.33, 95% CI 1.83-2.98; *P*<.001; *χ*^2^=6.0; *I*^2^=67%; *P*=.05).

For continuous measures, pooled analyses using change-from-baseline data under random-effects models showed nonsignificant but directionally favorable reductions in systolic and diastolic BP among participants receiving nurse-led telecare. The MD for systolic BP was –4.83 mm Hg (95% CI –12.51 to 2.85; *I*^2^=92%; *P*=.22) and for diastolic BP was –6.41 mm Hg (95% CI –13.76 to 0.93; *I*^2^=97%; *P*=.09).

Leave-one-out sensitivity analyses by delivery modality and follow-up duration yielded similar directions of effect without materially changing heterogeneity.

LDL-C also showed a small and statistically nonsignificant difference between the intervention and control groups (MD 0.01, 95% CI –0.08 to 0.09; *I*^2^=97%; *P*=.90), with wide confidence and prediction intervals reflecting substantial between-study variability.

### Psychological Burden

Of the 9 included studies, 4 (44.44%) examined psychological outcomes, specifically depressive symptoms and anxiety. Two (22.22%) studies reported no significant improvements in depression scores following telecare interventions [25,32], while 2 (22.22%) others reported significant reductions in depressive and anxiety symptoms [27,29]. Notably, Boter and HESTIA Study Group [27] reported anxiety outcomes using median differences derived from a nonparametric analysis, which were not directly comparable to studies reporting MDs. The mixed results may reflect differences in intervention content, frequency, participant characteristics (eg, baseline distress), and outcome measurement tools.

### QoL Results

A total of 2 (22.22%) of the 9 studies evaluated QoL. Boter and HESTIA Study Group [27] reported a significant improvement only in the “role emotional” domain, with no significant effects in other domains. Another study found no significant impact of telecare on QoL [32]. Overall, evidence on QoL remained inconclusive, with limited studies and inconsistent findings.

### Medication Adherence

Only 1 (11.11%) of the 9 studies assessed medication adherence [26]. The trial reported no statistically significant improvement following telecare compared to usual care. The authors noted that many control participants were already using medication aids (eg, dosettes), which may have limited the scope for additional benefit.

### Health Care Service Use

Of the 9 included studies, 3 (33.33%) did not find that the intervention increased the number of times stroke survivors used primary care consultations as well as provider visits [27,29,31]. However, the intervention could significantly reduce the number of emergency department visits and hospital admissions [31].

### Stroke Recurrence Rate

A total of 2 (22.22%) of the 9 studies reported recurrent stroke outcomes, but only 1 (11.11%) study included this as a formal end point in statistical testing [32]. The study found no significant difference in recurrence rates between telecare and control groups over 12 months. The other study by MacKenzie et al [26] reported recurrence descriptively, with similarly low event rates across groups.

### Survivor Functioning and Coping

Mou et al [29] found that teleconsultation could significantly improve coping with the disease among stroke survivors. In addition, teleconsultation could also significantly reduce urinary tract problems and the number of falls among stroke survivors [30].

### Risk of Bias

**Figure 3:**
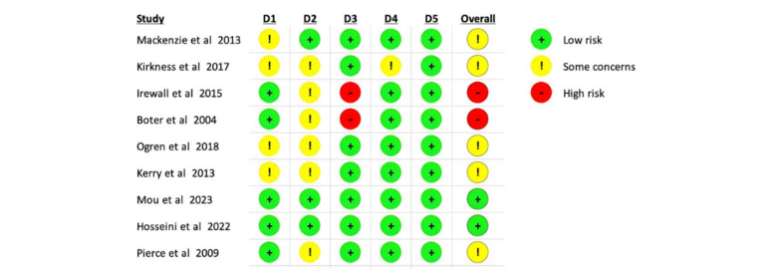
Risk of bias.

A summary of the risk of bias in the studies is shown in Figure 3 [25-33]. The risk of bias on the “selection of the reported result” dimension was rated as “low risk” for all studies. A total of 8 (88.89%) of the 9 studies were rated as “low risk” for the dimension “measurement of the outcome,” and 1 (11.11%) study was rated as “some concerns” because the outcome assessor was aware of the type of intervention the participants received, which could affect the measurement of the outcome. The risk of bias for the “missing outcome data” dimension was rated as “low risk” for 7 (77.78%) studies. However, 2 (22.22%) studies were rated as “high risk” because they did not provide key information on missing data. Due to the nature of telecare consultations, it was difficult to blind the intervention providers or participants; hence, 6 (66.67%) studies were rated as “some concerns” in the “deviation from the intended interventions” dimension, and only 3 (33.33%) studies were rated as “low risk.” In the “randomization process” dimension, 4 (44.44%) studies were rated as “some concerns” because some of the key variables differed between the intervention group and control group in the baseline characteristics, and the remaining 5 (55.56%) studies were rated as “low risk.”

### Certainty of Evidence (GRADE)

According to the GRADE framework, certainty ranged from moderate for the proportion achieving target BP to low or very low for most other outcomes due to risks of bias, inconsistency (including substantial heterogeneity), imprecision (small samples or few events), and indirectness (short follow-up or high baseline adherence). Detailed ratings and downgrading rationales are summarized in Multimedia Appendix 4 [25-29,31-33].

## Discussion

### Principal Findings

On the basis of 9 studies involving 2524 participants, this systematic review and meta-analysis indicates that nurse-led telecare may contribute to improved BP management, better coping, and reduced hospital admissions among patients who have experienced a stroke. However, pooled effects on continuous outcomes did not reach statistical significance, and substantial heterogeneity was observed. Its impact on psychological well-being and QoL remains inconclusive due to mixed findings and limited evidence. Numerous studies have established that effective BP control is associated with a lower risk of recurrent stroke [34]. In our review and meta-analysis, the proportion of stroke survivors achieving their target BP was significantly higher in the intervention group; however, pooled analyses of systolic and diastolic BP change showed nonsignificant yet directionally favorable reductions. These findings are generally consistent with findings from a previous review [35], but the wide confidence and prediction intervals highlight considerable uncertainty. From a behavioral change perspective, the capability, opportunity, motivation, and behavior (COM-B) model suggests that such improvements may arise from enhanced capability through targeted education and self-monitoring skills, increased opportunity via removal of travel and mobility barriers, and strengthened motivation through regular feedback and goal reinforcement. Parallelly, the self-efficacy theory by Bandura [36] explains how personalized feedback, problem-solving support, and encouragement during telecare sessions may increase patients’ confidence in managing their cardiovascular risk, thereby promoting sustained adherence to BP control behaviors. With respect to lipids, the pooled effect on LDL-C was small and statistically nonsignificant (directionally favoring the intervention). Using typical LDL-C SDs in secondary prevention cohorts (~25-35 mg/dL) likely corresponds to an average absolute reduction of approximately 3 to 5 mg/dL. Given the wide confidence and prediction intervals and the small absolute change, this effect is unlikely to be clinically meaningful at the individual level. Nonetheless, even modest LDL-C improvements, when combined with the larger gains in BP control and scaled across high-risk populations, may contribute to incremental vascular risk reduction.

However, we observed substantial heterogeneity (*I*^2^>90%) in the pooled results for systolic and diastolic BP. This level of variability indicates genuine clinical and methodological diversity rather than random error; therefore, the results should be interpreted with caution. Several factors likely contributed to this variability. Intervention modality differed substantially, ranging from telephone-only follow-ups to telemonitoring or app-based platforms. Interventions incorporating remote physiological monitoring with structured feedback may have enabled more rapid clinical adjustments [32], whereas telephone-only models often relied on self-reported values and thus may have been less precise. Follow-up duration also varied widely, from 1 to 36 months, with shorter studies capturing early gains and longer studies influenced by adherence fatigue, comorbidity progression, or care delivery changes. Baseline BP control further moderated outcomes; trials with participants already meeting target BP had less scope for improvement, while those with poorer baseline control tended to report larger reductions. In addition, the health care system context and scope of nursing autonomy appeared influential. In systems where nurses could directly adjust antihypertensive therapy, BP improvements were generally greater. In contrast, settings requiring physician referral for medication changes introduced delays that may have attenuated benefits. Finally, participant characteristics, such as stroke severity, caregiver support, and digital literacy, likely influenced engagement and intervention effectiveness. Collectively, these variations underscore the importance of examining contextual and patient-level effect modifiers in future studies, ideally through stratified analyses to identify the telecare models most effective for poststroke BP control.

It is important to recognize that BP control is closely linked to medication adherence [37]. However, evidence on the impact of telecare interventions on medication adherence remains limited and inconclusive. MacKenzie et al [26] found no significant difference in medication adherence between the intervention and control groups. Notably, some control participants were already using dosettes, which may have diluted the observed effect. Furthermore, variation in medication burden among participants, particularly polypharmacy, could have confounded the adherence outcomes, as polypharmacy is known to reduce adherence [38]. This finding is consistent with other literature suggesting that telecare may have minimal additive effects in populations with high baseline adherence [39,40]. Conversely, previous systematic reviews have demonstrated that telecare may improve adherence by serving as a structured reminder system [41]. Modality may also matter; app- or SMS-based interventions may offer greater flexibility and user engagement compared to telephone calls. Future research should target populations with low baseline adherence and minimize variability in intervention delivery and participant characteristics to better evaluate the effects of telecare on medication-taking behavior.

The impact of telecare on psychological well-being among stroke survivors was inconclusive, with studies reporting both positive and null results. Some trials indicated reductions in depressive and anxiety symptoms [27,29], while others showed no significant improvements [25,32]. These discrepancies may stem from differences in intervention design, intensity, baseline psychological distress, and follow-up duration. In addition, some confounding factors, such as control group participants seeking external counseling, may have masked intervention effects [25]. While a previous meta-analysis reported no overall effect of telecare on depression [42], the included studies often targeted functional recovery rather than mental health directly. Nevertheless, telecare may still enhance psychological well-being by fostering structured self-assessment, providing personalized support, and increasing carer involvement. These elements can improve motivation, confidence, and perceived competence in stroke survivors [43-48].

Similarly, the effect of telecare on QoL remains uncertain. Only a few of the 9 studies assessed this outcome, with 1 (11.11%) reporting domain-specific improvements (eg, “role emotional”) [27], while another found no significant changes [32]. Methodological factors, such as imprecise measurement tools and limited intervention scope, may have limited the detection of QoL improvements. From a theoretical standpoint, the COM-B model suggests that while telecare may enhance capability and motivation for health behaviors, QoL improvements often require broader opportunities for social participation, physical activity, and functional independence—elements not consistently addressed in the included interventions. Likewise, self-efficacy theory implies that gains in confidence and self-management skills, though valuable, may not translate into QoL improvements without concurrent enhancements in physical function, community engagement, and emotional well-being. This may explain why QoL effects were inconsistent across studies and suggests that telecare’s impact on QoL could be strengthened by integrating multidisciplinary and socially focused components.

Our review highlights the positive effects of telecare on survivors’ functioning, disease coping, and reducing emergency department visits and rehospitalization rates. Supporting these findings, Hwang et al [49] suggest that telecare enables stroke survivors to develop proficiency with coping skills through practice, which helps them better deal with psychological problems. Within the COM-B framework, coping gains may reflect enhanced capability through skills training in self-care and symptom management and increased opportunity by ensuring timely access to professional guidance when new challenges arise. Self-efficacy theory further suggests that these interventions provide mastery experiences (successfully applying coping strategies), verbal persuasion (encouragement from nurses), and emotional support—all of which build confidence in self-management. This combination of increased capability and confidence likely explains the observed improvements in coping outcomes in several studies.

Health care service use is a critical outcome in evaluating the scalability and cost-effectiveness of nurse-led telecare interventions. The findings across the included studies were mixed. Ajčević et al [50] reported that implementing telecare during the early poststroke phase prevented acute complications, resulting in fewer emergency department visits. Similarly, Thomas et al [51] and Sharrief et al [52] found that telecare improved patients’ coping strategies and self-management skills, which facilitated early recognition of clinical deterioration and timely referrals, thereby potentially averting avoidable hospital admissions. These findings highlight telecare’s potential to function as an early warning and triage mechanism, helping to address issues before they escalate. In contrast, Markle-Reid et al [53] did not observe significant changes in health care use. This may be attributed to the high level of multimorbidity among participants, which could obscure intervention effects, as well as limitations in how the use outcomes were measured. For example, some studies only tracked hospital admissions, overlooking subtler forms of use, such as teleconsultation frequency or primary care visits.

Despite these inconsistencies, the potential of telecare to reduce emergency care and hospitalization by supporting proactive management of poststroke complications remains an important consideration for health care systems. Such interventions may also alleviate pressures on outpatient services, particularly in resource-constrained or rural settings. Future research should adopt more comprehensive and standardized metrics for health care use and explore subgroup effects (eg, by stroke severity or comorbidity burden) to better determine where telecare offers the greatest benefit.

While health care use showed some promise, telecare did not significantly impact stroke recurrence in the included studies. Only 1 (11.11%) of the 9 studies directly evaluated this outcome, reporting no significant differences between intervention and control groups [54]. Another study noted a low recurrence rate, likely due to a younger and less severely affected population [55]. However, most RCTs did not prioritize stroke recurrence as a primary outcome, underscoring the need for future studies to better assess this end point through adequately powered and longer-term designs.

Effective stroke rehabilitation is crucial to prevent recurrent cardiovascular events in stroke survivors. Given the chronic nature of stroke recovery, telecare is a viable alternative for long-term follow-up. Particularly, telephone consultations are the most commonly used mode of service delivery [7,26-30]. This flexible approach, unrestricted by time and geographic constraints, enables nurses to provide regular monitoring, education, and support. These benefits may contribute to better management of modifiable cardiovascular risk factors, promote sustainable lifestyle changes, and enhance therapeutic adherence—key components of successful poststroke care [56].

### Strengths and Limitations

This systematic review strictly adhered to the PRISMA 2020 statement guidelines. In addition, an extensive literature search involving 6 databases was included. Compared with previous review studies [42,57], we provided a more comprehensive evaluation of the effects of telecare by incorporating a broader range of outcome measures. However, this study had several limitations. First, some of the included studies exhibited risks of bias to some extent that may have influenced the findings. Second, our meta-analysis had limited statistical power, as only 4 (44.44%) of the 9 studies were eligible for pooling, and the heterogeneity displayed a high level. Third, we excluded studies not published in English or Chinese, which may introduce language bias and limit the comprehensiveness of our review. Fourth, because the sample overwhelmingly consisted of ischemic stroke or transient ischemic attack (≈93%) survivors with few hemorrhagic stroke survivors, the findings may not generalize to patients who have experienced hemorrhagic stroke. Fifth, most studies had comparably short follow-up periods. They varied from 1 to 12 months, with only 1 (11.11%) study extending to 36 months. Therefore, further research with a longer follow-up period is needed to evaluate the long-term effects of telecare. Sixth, the high heterogeneity observed in several outcomes raises caution in interpreting the pooled estimates. Although all included studies used consistent measurement units for certain outcomes (eg, mm Hg for BP), there was considerable variation in the specific outcome measures (eg, different depression or QoL scales) and in the intervention delivery methods (eg, telephone-only follow-up, telemonitoring, app-based platforms, or combinations). This variability likely reduced the direct comparability of findings across trials and limited the generalizability of our conclusions to all nurse-led telecare models or settings. Future studies should adopt more standardized outcome definitions, validated measurement tools, and clearly described intervention protocols to facilitate robust cross-study comparisons.

### Implications for Practice and Policy

Despite the heterogeneity of interventions and settings, this review offers several practical insights. First, nurse-led telecare appears most effective when focused on risk factor management, particularly for BP control, and when supported by regular monitoring and decision support systems. Clinicians should consider integrating telecare into poststroke care pathways, especially for patients with mobility limitations or those living in remote areas. Second, interventions delivered via mobile apps or messaging platforms may offer greater flexibility and engagement compared to telephone-only formats. Health systems should prioritize standardized protocols, training for telecare providers, and tailored interventions based on patients’ needs, such as mental health support or medication adherence coaching. Finally, implementation should be guided by infrastructure readiness, including digital access and caregiver support, to maximize equity and effectiveness. While the evidence base remains limited in some domains, telecare offers a scalable and patient-centered complement to traditional follow-up care.

### Conclusions

This review suggests that nurse-led telecare consultations tend to improve BP and lipid profiles and support coping strategies and may reduce acute health care use among community-dwelling stroke survivors. However, most continuous outcomes did not reach statistical significance, and substantial heterogeneity limits the certainty of these findings. The strength of evidence remains limited due to the small number of studies per outcome, high heterogeneity, and the underrepresentation of stroke recurrence as a primary end point. Most included studies also had short follow-up durations, limiting our understanding of long-term effects. While telecare appears to be a promising alternative for delivering poststroke care, more rigorous and long-term trials are needed to substantiate its effectiveness and guide clinical integration.

## Data Availability

Data sharing is not applicable to this study as no datasets were generated or analyzed during this study.

## Appendix

Multimedia Appendix 1 Search strategies.

Multimedia Appendix 2 Inclusion and exclusion criteria based on the population, intervention, comparison, outcomes, and study design framework.

Multimedia Appendix 3 Data extraction table.

Multimedia Appendix 4 Grading of Recommendations Assessment, Development, and Evaluation evidence profile.

Multimedia Appendix 5 PRISMA checklist.

## Abbreviations

APN: advanced practice nurse
BP: blood pressure
COM-B: capability, opportunity, motivation, and behavior
GRADE: Grading of Recommendations Assessment, Development, and Evaluation
LDL-C: low-density lipoprotein cholesterol
MD: mean difference
OR: odds ratio
PRISMA: Preferred Reporting Items for Systematic Reviews and Meta-Analyses
QoL: quality of life
RCT: randomized controlled trial
