# Supplementary material for: The Effectiveness of Nurse-Led Telecare Consultations Among Patients Who Have Experienced a Stroke: Systematic Review and Meta-Analysis
Source: J Med Internet Res. 2025 Nov 27;27:e74149. doi: 10.2196/74149 (PMC12699255; doi:10.2196/74149)
Supplement: Multimedia Appendix 1 [file jmir_v27i1e74149_app1.docx]

## Multimedia Appendix 2

Searching Strategies

1. PubMed（20586）

| Searching Strategy | (((((("Telecommunications"[Mesh]) OR "Telemedicine"[Mesh]) OR "Remote Consultation"[Mesh]) OR "Distance Counseling"[Mesh]) OR "Telenursing"[Mesh]) OR "Telerehabilitation"[Mesh]) OR (technolog* OR telerehabilitation OR e-rehabilitation OR telecare OR telemedicine OR telehealth OR telecommunication OR telemonitor* OR telenursing OR tele-nursing OR ehealth OR e-health OR mhealth OR m-health OR telecoaching OR gerontechnology OR videoconferenc* OR teleconferenc* OR internet OR computer OR mobile OR phone OR smartphone OR telephone OR tablet OR email OR e-mail OR SMS OR apps OR applications OR "social media" OR wireless OR virtual OR remote OR distant)  AND  ("Stroke Rehabilitation"[Mesh]) OR (post-stroke OR "after stroke" OR "stroke recurrence" OR "stroke survivor*" OR "outpatient stroke" OR "stroke aftercare")  AND  ((("Nurses"[Mesh]) OR "Nurse Practitioners"[Mesh]) OR "Nurse Specialists"[Mesh]) OR (nursing or nurs* or nurse-led or ''nurse led' or nurse-based or 'nurse based' or interdisciplin* or multi-disciplin* or multidisciplin*) |
| --- | --- |
| Filter Applied | - Article Type: Randomized Controlled Trial |

2. MEDLINE (via Ebscohost)（1081）

| Searching Strategy | (((exp Remote Consultation/ or exp Videoconferencing/ or exp Telemedicine/) OR (exp Social Media/)) OR (technolog* or telerehabilitation or e-rehabilitation or telecare or telemedicine or telehealth or telecommunication or telemonitor* or telenursing or tele-nursing or ehealth or e-health or mhealth or m-health or telecoaching or gerontechnology or videoconferenc* or teleconferenc* or internet or computer or mobile or phone or smartphone or telephone or tablet or email or e-mail or SMS or apps or applications or social media or wireless or virtual or remote or distant)))  AND  ((exp Stroke Rehabilitation/) OR (post-stroke or 'after stroke' or 'stroke recurrence' or 'stroke survivor*' or 'outpatient stroke' or 'stroke aftercare'))  AND  ((exp Nurses/) OR (nurs* or nurse-led or ''nurse led' or nurse-based or 'nurse based' or interdisciplin* or multi-disciplin* or multidisciplin*)) |
| --- | --- |
| Filter Applied | - Scholarly (Peer Reviewed) Journals - Publication Type: Randomized Controlled Trial |

3. CINAHL (via Ebscohost)（352）

| Searching Strategy | (((((MH "Telecommunications+") OR (MH "Telecommuting") OR (MH "Teleconferencing") OR (MH "Telehealth+") OR (MH "Telefacsimile") OR (MH "Videoconferencing+") OR (MH "Text Messaging+") OR (MH "Telephone+") OR (MH "Telenursing+") OR (MH "Telemedicine+") OR (MH "Email") OR (MH "Instant Messaging") OR (MH "Interactive Voice Response Systems") OR (MH "Internet+") OR (MH "Voice Mail") OR (MH "Wireless Communications") OR ((MH "Remote Consultation") OR (MH "Remote Access to Information") OR (MH "Videoconferencing+")) OR ((MH "Nurse Consultants+") OR (MH "Telephone Consultation (Iowa NIC)")) OR ((MH "Nurse Consultants+") OR (MH "Telephone Consultation (Iowa NIC)"))) OR (technolog* OR telerehabilitation OR e-rehabilitation OR telecare OR telemedicine OR telehealth OR telecommunication OR telemonitor* OR telenursing OR tele-nursing OR ehealth OR e-health OR mhealth OR m-health OR telecoaching OR gerontechnology OR videoconferenc* OR teleconferenc* OR internet OR computer OR mobile OR phone OR smartphone OR telephone OR tablet OR email OR e-mail OR SMS OR apps OR applications OR "social media" OR wireless OR virtual OR remote OR distant))))  AND  "stroke rehabilitation" or "stroke recovery" or post-stroke OR "after stroke" OR "stroke recurrence" OR "stroke survivor*" OR "outpatient stroke" OR "stroke aftercare" or "post stroke"  AND  (((MH "Practical Nurses") OR (MH "Primary Care Nurse Practitioners") OR (nurse or nurses or "Nurse Practitioners" or "Nurse Specialists" or nursing or nurs* or nurse-led or ''nurse led' or nurse-based or 'nurse based' or interdisciplin* or multi-disciplin* or multidisciplin*)) |
| --- | --- |
| Filter Applied | - Peer Reviewed - Randomized Controlled Trial |

4. EMBASE（456）

| Searching Strategy | (((('telecommunication'/exp OR 'telehealth'/exp OR 'e-health' OR 'ehealth' OR 'tele-health' OR 'telehealth' OR 'telemedicine'/exp OR 'tele medicine' OR 'virtual medicine' OR 'teleconsultation'/exp OR 'long distance consultation' OR 'remote consultation' OR 'tele-consultation' OR 'telephone consultation' OR 'telephone-based consultation' OR 'electronic consultation'/exp OR 'video consultation'/exp OR 'telemedicine video-consultation' OR 'videoconsultation') OR ('telerehabilitation'/exp OR 'e-rehabilitation' OR 'remote rehabilitation' OR 'tele-rehabilitation' OR 'virtual rehabilitation')) OR ('telecare'/exp OR 'e-care' OR 'e-health care' OR 'e-healthcare' OR 'tele-care' OR 'virtual care' OR 'virtual health care' OR 'virtual healthcare'))) OR (internet OR computer OR mobile OR phone OR smartphone OR telephone OR tablet OR email OR e-mail OR SMS OR apps OR applications OR "social media" OR wireless OR virtual OR remote OR distant))))  AND  ((“stroke rehabilitation” OR "post-stroke" OR "after stroke" OR "stroke recurrence" OR "stroke survivor*" OR "outpatient stroke" OR "stroke aftercare"))  AND  (('nurse'/exp OR 'advanced practice nurse'/exp OR 'nurse practitioner'/exp OR 'nurse specialist'/exp) OR (nurs* OR 'nurse led' OR 'nurse based' OR interdisciplin* OR 'multi disciplin*' OR multidisciplin*)) |
| --- | --- |
| Filter Applied | - Article type: Article - Randomized Controlled Trial |

5. PsycINFO (via ProQuest)（82）

| Searching Strategy | (((DE "Telecommunications Media" OR DE "Radio" OR DE "Telephone Systems" OR DE "Television" OR DE "Wireless Technologies" OR DE "Teleconferencing" OR DE "Videoconferencing" OR DE "Teleconsultation" OR DE "Telemedicine" OR DE "Online Therapy" OR DE "Teleconferencing" OR DE "Teleconsultation" OR DE "Telepsychiatry" OR DE "Telepsychology" OR DE "Telerehabilitation" OR DE "Telerehabilitation") OR (DE "Teleconsultation")) OR ("Remote Consultation" OR "Remote Access to Information" OR "Nurse Consultants+" OR MH "Nurse Consultants+" OR "Telephone Consultation" OR technolog* OR telerehabilitation OR e-rehabilitation OR telecare OR telemedicine OR telehealth OR telecommunication OR telemonitor* OR telenursing OR tele-nursing OR ehealth OR e-health OR mhealth OR m-health OR telecoaching OR gerontechnology OR videoconferenc* OR teleconferenc* OR internet OR computer OR mobile OR phone OR smartphone OR telephone OR tablet OR email OR e-mail OR SMS OR apps OR applications OR "social media" OR wireless OR virtual OR remote OR distant)))  AND  ("stroke rehabilitation" or "stroke recovery" or post-stroke OR "after stroke" OR "stroke recurrence" OR "stroke survivor*" OR "outpatient stroke" OR "stroke aftercare" or "post stroke")  AND  ((DE "Nurses" OR DE "Nursing") OR ("Practical Nurses" OR "Primary Care Nurse Practitioners" OR nurse or nurses or "Nurse Practitioners" or "Nurse Specialists" or nursing or nurs* or nurse-led or ''nurse led' or nurse-based or 'nurse based' or interdisciplin* or multi-disciplin* or multidisciplin*))  AND  ((“randomi?ed control* trial” OR RCT OR “control* trial” OR “random* allocat*” OR “random* assign*” OR “randomi?ed control* clinical trial” OR “clinical control* trial” OR “randomi?ed control* study” OR randomi?ed)) |
| --- | --- |
| Filter Applied | - Peer reviewed |

6. Cochrane Central Register of Controlled Trials (CENTRAL)（308）

| Searching Strategy | #1 MeSH descriptor: [Telecommunications] explode all trees  #2 ("telecommunications"):ti,ab,kw OR (technolog* OR telerehabilitation OR e-rehabilitation OR telecare OR telemedicine OR telehealth OR telecommunication OR telemonitor* OR telenursing OR tele-nursing OR ehealth OR e-health OR mhealth OR m-health OR telecoaching OR gerontechnology OR videoconferenc* OR teleconferenc* OR internet OR computer OR mobile OR phone OR smartphone OR telephone OR tablet OR email OR e-mail OR SMS OR apps OR applications OR “social media” OR wireless OR virtual OR remote OR distant):ti,ab,kw  #3 #1 OR #2  #4 MeSH descriptor: [Stroke Rehabilitation] explode all trees  #5 ("stroke rehabilitation"):ti,ab,kw OR ("stroke recovery" or "post-stroke" OR "after stroke" OR "stroke recurrence" OR "stroke survivor" OR "outpatient stroke" OR "stroke aftercare" or "post stroke"):ti,ab,kw  #6 #4 OR #5  #7 MeSH descriptor: [Nursing] in all MeSH products  #8 ("nursing"):ti,ab,kw AND ("nurse" OR "nurses" OR "Practical Nurses" OR "Primary Care Nurse Practitioners" OR "Nurse Practitioners" or "Nurse Specialists" or nurse-led or ''nurse led' or nurse-based or 'nurse based' or interdisciplinary or multi-disciplinary or multidisciplinary):ti,ab,kw  #9 #7 OR #8  #10 #3 AND #6 AND #9 |
| --- | --- |
| Filter Applied | - CENTRAL Trials only - Trial |
