# Supplementary material for: The Effectiveness of Nurse-Led Telecare Consultations Among Patients Who Have Experienced a Stroke: Systematic Review and Meta-Analysis
Source: J Med Internet Res. 2025 Nov 27;27:e74149. doi: 10.2196/74149 (PMC12699255; doi:10.2196/74149)
Supplement: Multimedia Appendix 2 [file jmir_v27i1e74149_app2.docx]

Table 1 Inclusion and Exclusion Criteria Based on PICOS Framework

| PICOS Domain | Inclusion Criteria | Exclusion Criteria |
| --- | --- | --- |
| Population | Stroke survivors aged 18 or above, living independently in the community (i.e., not in healthcare facilities). | Patients who were hospitalised or residing in assisted residential care settings, such as nursing homes or elderly homes. |
| Intervention | Nurse-led telecare consultation delivered via telephone, mobile health (mHealth) apps, videoconferencing, text messaging, or social media. Nurses must provide ≥50% of the care. | — |
| Comparison | Usual care. | — |
| Outcomes | At least one of the following: blood pressure, psychological burden, quality of life, medication adherence, healthcare service use, stroke recurrence rate, survivor functioning, and coping. | — |
| Study Design | Randomised controlled trials (RCTs). | Scoping reviews, narrative reviews, conference abstracts, articles without abstracts, or those without full text. |
| Additional Criteria | Articles published in English or Chinese. | Articles published in languages other than English or Chinese. |
