# Supplementary material for: The Effectiveness of Nurse-Led Telecare Consultations Among Patients Who Have Experienced a Stroke: Systematic Review and Meta-Analysis
Source: J Med Internet Res. 2025 Nov 27;27:e74149. doi: 10.2196/74149 (PMC12699255; doi:10.2196/74149)
Supplement: Multimedia Appendix 3 [file jmir_v27i1e74149_app3.docx]

## Multimedia Appendix 3

Data extraction table

| **Author**  **(Year of publication)** | **Study Location** | **Study Population** | **Provider** | **Duration** | **Intervention Group (IG)** | | **Control Group (CG)** | **Data Collection Timepoint** | **Outcome Variables** | **Outcome Measures** | **Result** |
| --- | --- | --- | --- | --- | --- | --- | --- | --- | --- | --- | --- |
|  |  |  |  |  | **Technological**  **(a) Delivery mode**  **(b) Content**  **(c) Frequency/Duration** | **Non-technological**  **(a) Delivery mode**  **(b) Content**  **(c) Frequency/Duration** |  |  |  |  |  |
| MacKenzie et al.  (2013) ^1^ | Canada | Clients with hypertension and probable TIA/ confirmed stroke in addition to deficits in cognition, or less than 100% medication self-efficacy, and/or any self-reported non-adherence to medication  (n=56, >65 years: 58.9% )  **IG**: n=29  Mean Age: NA  **CG:** n=27  Mean Age: NA | Nurses | 6 months | **Nurse-led case management program**  (a) Telephone calls  (b) Lifestyle changes, medication adherence counselling, home BP monitoring, medication dosettes administration  (c) Monthly | **Stroke physician specialist** (a) Clinic visits  (b) Assessment, treatment of hypertension with simplification of medication regimens where indicated  (c) NA | (1) Same assessment provided by stroke physician specialist  (2) Initiation and titration of BP medication  (3) Adherence and risk factor counselling at clinic visits  (4) Follow-up  by family physicians | T0: baseline  T1: 6 months | 1. Blood pressure  2. Medication self-efficacy  3. Medication adherence  4. Recurrence of probable TIA/stroke | 1. BPTru automated equipment  2. Self-developed seven-point Likert scale  3. Self-report of number of missed pills and community pharmacist review of participant prescription  renewal patterns  4. Based on SPC and  hospital re-admission documentation | (1) The decrease in SBP and DBP were not significant  [Decrease in SBP (mmHg): (IG) -7.2 (165.0) vs (CG) 16.8 (20.0); P=0.457]  [Decrease in DBP (mmHg): (IG) -22.2 (17.1) vs (CG) 8.1 (11.9); p=0365]  (2) An overall trend for more patients in the IG than in the CG achieved the target values for SBP, but not significant.  [(IG) 58.6 vs (CG) 37.0%; p = 0.11]  (3) Change in medication self-efficacy, change in missed pills, and change in % adherence did not differ between groups  [(IG) -34.8±184.7 vs (CG) -110.5±317.8; p=0.28]  [(IG) -34.0±185.6 vs (CG) -36.9±192.3; p=0.95]  [(IG) -45.1±185.4 vs (CG) 36.0±198.2; p=0.15] |
| Kirkness  et al.  (2017) ^2^ | US | Both ischemic and haemorrhagic stroke survivors with depression  (n=100)  **IG-telephone**: n=37  Mean Age: 61.7  **IG-in person**: n=35  Mean Age:58.5  **CG:** n=28  Mean Age: 60.7 | Psychosocial nurse practitioner therapist | 1 year | **Telephone Training session**  (a) Telephone calls  (b) Cognitive behavioural therapy  for depression after stroke  (c) 6 sessions, average 26 min verses 38 min each session | **Education session**  (a) face to face  (b) discussed goals and expectations of each session, and learned how to fill out the homework  Sections  (c) once  **Participant manuals**  (b) NA  **Prescription**  (b) antidepressants prescribed by the participant’s usual care provider | (1) reported on progress at follow up visits in their homes from the research nurses  (2) Same antidepressants prescription | T0: baseline  T1: 8 weeks  T2: 21 weeks  T3: 12 months | 1. Depressive symptom  2. Stroke impact  3. Perceived recovery | 1. Hamilton Rating Scale for Depression  2. Stroke impact scale  3. Percent perceived recovery | (1) The reduction in HDRS score was not significant between telephone IG and CG at 8,21 weeks and 12 months    (2) Remission in HDRS score was not significant between telephone IG and CG at 8,21 weeks and 12 months |
| Irewall  et al.  (2015) ^3^ | Sweden | Patients diagnosed with an acute stroke or TIA (n=484)  (Mean Age: 70.8±10.7)  **IG:** n=241  Mean Age: 71.5±11.1  **CG**: n=243  Mean Age: 70.1±10.4 | Nurses | 12 months | **Telephone-based counselling**  (a) Telephone  (b) telephone-based lifestyle counselling and assessment of pharmacological treatment  (c) Once a month | **Medication Adjustment**  (b) Study physician would adjust the medication if BP or LDL was higher than anticipated | (1) Local standard  procedures  (2) BP and LDL-C values were forwarded to patient’s general practitioners | T0: baseline  T1: 12 months | 1. Mean difference in systolic blood pressure (SBP)  2. Mean difference in diastolic blood  pressure (DBP)  3. Mean difference in LDL-C | 1. Clinical measurements  2. Clinical measurements  3. Calculated from the serum concentrations of cholesterol and fasting triglycerides using the Friedewald formula | (1) The mean difference in SBP and DBP between groups are significant  [Mean SBP mmHg 3.3 (95% CI 0.3 to 6.3)]  [Mean DBP mmHg 2.3 (95% CI 0.5 to 4.2)]  [Mean LDL-C mmol/L 0.3 (95% CI 0.1 to 0.4)]  (2) Significantly more patients in the IG than in the CG achieved the target values for SBP and LDL-C.  [(IG) 68.5 vs (CG) 56.8%; p = 0.008]  [(IG) 69.7% vs (CG) 50.4%; p < 0.001] |
| Boter  et al.  (2004) ^4^ | Netherland | transient ischemic attack or ischemic stroke, primary intracerebral hemorrhage,  or subarachnoid hemorrhage  (n=536)  (Mean Age: NA)  **IG**: n=263  (Analyzed: 231)  Mean Age, IQR: 66 (52, 76)  **CG**: n=273  (Analyzed: 255)  Mean Age: 63 (51-74) | Nurses | 6 months | **Outreach care program**  (a) Telephone calls  (b) Support patients and carers according to their individual needs by giving information and reassurance or, when the presented problem required additional care or exceeded the nurses’ expertise, advised patients or carers to contact the general practitioner  (c) 3 telephone calls | **Checklist Development**  (b) A standardized  checklist containing risk factors for stroke, consequences of stroke, and unmet needs for stroke. Nurses kept a detailed record of all contacts, which allowed referring to previously raised needs, and applied interventions  **Brochures**  (b) Information on stroke management | Standard care | T0: baseline  T1: 6 months | 1. Satisfaction with the care received  2. Quality of life  3. Anxiety and depression  4. Sense of competence  5. Discrepancies in social support measured by the subscales problem-oriented emotional support and social companionship | 1. Satisfaction-With-Stroke-Care questionnaire (SASC-19)  2. Short Form 36 (SF-36)  3. Hospital Anxiety and Depression Scale (HADS)  4. Sense of Competence Questionnaire (SCQ)  5. Social Support List–Discrepancies (SSL-D) | (1) Patients in the IG had better scores on the SF-36 domain “Role Emotional” than controls  [mean difference 7.9 [95% CI, 0.1 to 15.7]  (2) No statistically significant differences were found on patients’ satisfaction with the care received and other domains of quality of life  (3) Patients in the IG used fewer rehabilitation services  [relative risk, 0.66 (0.44 to 1.00)]  (4) Patients in the IG had lower anxiety scores  [median difference 1 (0.19 to 2.79), based on non-parametric estimation]. The result was therefore not pooled with studies reporting mean differences. |
| Ögren  et al.  (2018) ^5^ | Sweden | All patients treated with an intracerebral hematoma (ICH), ischemic stroke (IS) or TIA  (n=660)  (Mean Age: 69.6)  **IG:** n=320  Mean Age: 69.9  **CG**: m=340  Mean Age: 69.3 | Nurses | 36 months | **Telephone-based follow-up**  (a) Telephone calls  (b) Telephone-based counselling. Patients who did not meet the target values received additional follow-up, including lifestyle counselling to reach their treatment goals  (c) One month after discharge and yearly thereafter | **Medical treatment adjustment**  (b) A study physician was consulted to assess and adjust the medical treatment when the participants did not achieve the set target for LDL-C and/or BP | 1. Treatment was generally initiated  in-hospital and after discharge  2. Received secondary  preventive care according to local standards, most often  by each patient’s general practitioner | T0: baseline  T1: 1 month  T2: 12 months  T3: 24 months  T4: 36 months | 1. Systolic blood pressure (SBP)  2. Diastolic blood  pressure (DBP)  3. LDL-C  4. Proportion of patients reaching set targets for these variables | 1. Clinical measurements  2. Clinical measurements  3. Clinical measurements  4. Clinical measurements | (1) The mean SBP value was significantly lower in the IG than CG (36 months)  [IG vs CG 6.1 mmHg lower, 95% CI 3.6–8.6,  p < 0.001]  (2) The mean DBP value was significantly lower in the IG than CG (36 months)  [IG vs CG 3.4 mmHg lower, 95% CI 1.8–5.1,  p < 0.001]  (3) The mean LDL-C value was significantly lower in the IG than CG (36 months)  [IG vs CG 0.3 mmol/L lower, 95% CI 0.2–0.5,  p < 0.001]  (4) A larger proportion of IG reached the treatment goal for SBP, DBP and LDL-C than CG (36 months)  [SBP: (IG) 79.4% vs (CG) 55.3%, p < 0.001]  [DBP: (IG) 90.3% vs (CG) 77.9%, p < 0.001]  [LDL-C: (IG) 69.3% vs (CG) 48.9%, p < 0.001] |
| Kerry  et al.  (2013) ^6^ | UK | Had a history of stroke or transient ischemic attack within the 9 months before enrolment and had hypertension  (n=381)  (Mean Age: 72, 30-94)  **IG**: n=194  Mean Age: 72.6 ± 11.4  **CG:** n=187  Mean Age: 71.1 ± 12.6 | Nurses | 12 months | **Home monitoring**  (a) Telephone calls  (b) Check technique and review blood pressure readings  (c) One week, 3 months and 9 months (and at 6 months if the reading taken by the research assistant was elevated) after the start of the intervention | **Blood pressure diary**  (b) record BP  (c) Take 3 readings, 1 minute apart, daily for the first week, then weekly  **Referral to physicians**  (b) Participants whose blood pressure was consistently above target were advised to see their physician and to take their booklet | 1. Received usual care from their physician but no intervention from the nurse  2. Study administrator telephoned control participants after 3 and 9 months to check on their well-being, but no advice on blood pressure was given | T0: baseline  T1: 6 months  T2: 12 months | 1. Change in mean SBP after 12 months  2. Change in mean SBP after 6 months  3. Change in mean DBP  4. Change on number of antihypertensive drugs  5. Treatment changes  6. Primary care consultations  7. Change in EQ- 5D  8. Change in FEAR scores  9. Recurrence of stroke | 1. Clinical measurements  2. Clinical measurements  3. Clinical measurements  4. Participants’ reports  5. Participants’ reports  6. Family practice electronic medical records  7. Euro-Qol 5-dimension (EQ-5D) index  8. Frequency of anxiety, enduring nature of anxiety, alcohol or sedative use, restlessness, or fidgeting Questionnaire (FEAR)  9. Medical record | (1) The fall in SBP from baseline did not differ significantly between the groups  [Adjusted mean difference 0.3 mmHg, 95% CI –3.6 to 4.2 mm Hg]  (2) More patients in the IG than in the CG having changes to antihypertensive treatment during the trial period  [(IG) 60.1% vs (CG) 47.6%, p = 0.02]  (3) There was no difference between the groups in the number of medications per patient.  (4) There was no significant difference in quality of life and psychological burden between the groups  (5) The number of primary care consultations reported by participants did not differ significantly between the groups  [(IG) 5.2±4.6 per patient vs (CG) 5.4±5.3 per patient]  (6) The rate of recurrent stroke did not differ significantly between the groups  [(IG) 6.1% vs (CG) 8.1%] |
| Mou  et al.  (2023) ^7^ | HK | Stroke survivors  (n=162)  (Mean Age: 56.07 ± 11.17)  **IG**: n=81  Mean Age: 54.63 ± 11.80  **CG**: n=81  Mean Age: 57.52 ± 10.37 | Nurse | 3 months | **FDPEI programme**  (a) Telephone counselling  (b) Encourage the dyads to identify post-stroke distress or difficulties and motivating them to cope with these difficulties or problems by providing regular post-discharge counselling.  (c) Four weekly telephone calls, each lasting approximately 30 mins | **Structured face-to-face education**  (a) Face to face  (b) stroke-related knowledge, rehabilitation, self-care skills, and psychotherapeutic  techniques to help them prepare for the transition from hospital to home  (c) 3 sessions completed within a week of hospital pre-discharge and each session was 60 min | 1. Usual care focused on stroke survivors, including medical treatments and care provided by doctors and  nurses, and rehabilitation services based on the patient's condition.  2. Survivors and/or their family caregivers received one or two  brief health education lessons on post-stroke lifestyle management,  which was provided by nurses or other healthcare professionals | T0: baseline  T1: 1 month after discharge  T2: 3-month follow up | 1. Survivor functioning  2. Dyads’ coping  3. Dyads’ family functioning  4. Dyads' perceived dyadic relationship  5. Dyads' depressive symptoms  6. Dyads' anxiety symptoms  7. stroke survivors' utilisation of healthcare services at one month after discharge (T1) and 3-month follow-up (T2) | 1. Stroke Impact Scale (SIS) version 3.0  2. Family Crisis Oriented Personal Evaluation Scale  3. General Functioning of Family Assessment Device  4. Mutuality Scale  5. Patient Health Questionnaire-9 (PHQ-9)  6. Generalised Anxiety Disorder Scale-7  7. Two dichotomous questions | (1) There was no significant difference between IG and CG in survivor functioning except in emotion domain at T1  [(IG) 78.74±16.49 vs (CG) 73.01±18.91, β = 7.22, p = 0.015]  (2) The IG demonstrated significantly greater survivors' depressive symptoms at T1 and T2  [T1: (IG) 3.75±3.61 vs (CG) 4.97±3.66, β = −1.56, p = 0.007]  [T2: (IG) 3.43±4.08 vs (CG) 5.24±5.02, β = −2.06, p = 0.005]  (3) The IG demonstrated significantly greater dyadic relationship at T1 and T2  [T1: (IG) 3.01±0.56 vs (CG) 2.79±0.59, β = 0.26, p = 0.012]  [T2: (IG) 3.10±0.67 vs (CG) 2.88±0.70, β = 0.27, p = 0.022]  (4) The IG demonstrated significantly greater on survivor coping at T2  [(IG) 96.31±12.03 vs (CG) 88.54±16.76, β = 6.73, p = 0.008] |
| Hosseini  et al.  (2022) ^8^ | Iran | Acute brain stroke discharged from neurological wards  (n=72)  (Mean Age: NA)  **IG**: n=36  Mean Age: 65.89±12.56  **CG**: n=36  Mean Age: 69.72±14.59 | Nurse | 1 month | **Remote training and counselling**  (a) Telephone calls  (b) Instructions to reduce the caregiver burden of care and prevent pressure ulcers, falls, and urinary tract problems in addition to routine training in the hospital  (c) At least 3 phone calls in the first week after discharge.  **Remote counselling and telephone follow-up**  (a) Telephone calls  (b) Counselling and telephone follow-up were performed based on patients’ needs  (c) In the next 3 weeks after discharge. Each call lasted between 15 to 20 minutes. The participants in the IG could call the nurse if they needed telephone counselling at any time of day and night | **Education session**  (a) Face to face before discharge  (b) a standard educational content to increase patient-caregiver awareness of preventative measures for the three most common stroke complications, including bedsores, urinary infections, and falls. Information about available medical and support centers was provided to caregivers | 1. Received only routine  discharge training provided by the department personnel  2. A brief summary of the teaching in the form of pamphlets | T0: Baseline  T1: 1 month | 1. Susceptibility to pressure ulcers  2. Susceptibility to falls  3. Signs and symptoms of urinary problems | 1. Braden scale  2. Morse scale  3. Urinary problem checklist | (1) The IG experienced remarkably fewer urinary problems than CG  [(IG) 0% vs (CG) 61.1%, P < 0.001]  (2) The IG experienced remarkably fewer falling than CG  [(IG) 0% vs (CG) 19.4%, P = 0.011] |
| Pierce  et al.  (2009) ^9^ | Toledo | First-time stroke survivors’ careers  (n=73)  (Mean Age: NA)  **IG**: n=36  Mean Age: 54.0±12.2  **CG**: n=37  Mean Age: 55.0±13.1 | Nurse (>50%), therapists, pharmacist, dietitian,  social worker and physician | 1 year | **Caring~Web Programme**  (a) Web-based  (b) Web-based education and support in home settings for carers of stroke survivors. four interrelated components for careers: (1) linked Web sites about stroke and caring; (2) customised educational information or tips specific to carers’ needs; (3) an email forum to ask a nurse specialist and a  rehabilitation team (therapists, pharmacist, dietitian, social worker and physician) any questions in private and (4) a non-structured email discussion amongst all participants facilitated by the nurse  (c) Trained telephone interviewers surveyed every 3 months. During this interview, as well as every 2 weeks, careers were queried about the use of healthcare services by the stroke survivor. |  | Non-Web user.  Usual care | T0: Baseline  T1: Month1-3  T2: Month 4-6  T3: Month 7-9  T4: Month 10-12 | 1. Health service use (visits to a provider and/or an emergency department, re-admissions to a hospital, or placements in a nursing home) | 1. Self-report | (1) No statistical differences were found between the groups in in the number of provider visits for survivors (1 year total)  [(IG) 460 vs (CG) 496, P=0,229]  (2) The number of emergency department visits by stroke survivors was significantly lower in IG than CG (1 year total)  [(IG) 27 vs (CG) 40, p=0.001]  (3) The number of hospital re-admissions related to the health of stroke survivors was significantly lower in IG than CG (1 year total)  [(IG) 14 vs (CG) 41, p=0.0005] |

Note: IQR indicates interquartile range. Boter et al. (2004) reported median differences derived from non-parametric analyses. These results were not directly pooled with studies reporting mean differences for the “psychological burden” outcome.

Reference:

1. Mackenzie G, Ireland S, Moore S, Heinz I, Johnson R, Oczkowski W, Sahlas D. Tailored interventions to improve hypertension management after stroke or TIA--phase II (TIMS II). Can J Neurosci Nurs. 2013;35(1):27-34. PMID: 23687780.

2. Kirkness CJ, Cain KC, Becker KJ, Tirschwell DL, Buzaitis AM, Weisman PL, McKenzie S, Teri L, Kohen R, Veith RC, Mitchell PH. Randomized trial of telephone versus in-person delivery of a brief psychosocial intervention in post-stroke depression. BMC Res Notes. 2017 Oct 10;10(1):500. doi: 10.1186/s13104-017-2819-y. PMID: 29017589; PMCID: PMC5633890.

3. Irewall A-L, Ögren J, Bergström L, Laurell K, Söderström L, Mooe T (2015) Nurse-Led, Telephone-Based, Secondary Preventive Follow-Up after Stroke or Transient Ischemic Attack Improves Blood Pressure and LDL Cholesterol: Results from the First 12 Months of the Randomized, Controlled NAILED Stroke Risk Factor Trial. PLoS ONE 10(10): e0139997.

[https://doi.org/10.1371/journal.pone.0139997](https://doi.org/10.1371/journal.pone.0139997 4)

[4](https://doi.org/10.1371/journal.pone.0139997 4). Boter H; HESTIA Study Group. Multicenter randomized controlled trial of an outreach nursing support program for recently discharged stroke patients. Stroke. 2004 Dec;35(12):2867-72. doi: 10.1161/01.STR.0000147717.57531.e5. Epub 2004 Oct 28. PMID: 15514186.

5. Ögren J, Irewall AL, Söderström L, Mooe T. Long-term, telephone-based follow-up after stroke and TIA improves risk factors: 36-month results from the randomized controlled NAILED stroke risk factor trial. BMC Neurol. 2018 Sep 21;18(1):153. doi: 10.1186/s12883-018-1158-5. PMID: 30241499; PMCID: PMC6148791.

6. Kerry SM, Markus HS, Khong TK, Cloud GC, Tulloch J, Coster D, Ibison J, Oakeshott P. Home blood pressure monitoring with nurse-led telephone support among patients with hypertension and a history of stroke: a community-based randomized controlled trial. CMAJ. 2013 Jan 8;185(1):23-31. doi: 10.1503/cmaj.120832. Epub 2012 Nov 5. PMID: 23128283; PMCID: PMC3537777.

7. Mou H, Lam SKK, Chien WT. The effects of a family-focused dyadic psychoeducational intervention for stroke survivors and their family caregivers: A randomised controlled trial. Int J Nurs Stud. 2023 Jul;143:104504. doi: 10.1016/j.ijnurstu.2023.104504. Epub 2023 Apr 17. PMID: 37149953.

8. Hosseini, A., Sharifi, N., dehghanrad, F., & Sharifipour, E. (2022). Effect of Telenursing on Caregiver Burden of Care and Incidence of some Complications in Patients with Acute Stroke Discharged from Neurological Wards: A Randomized Control Trial. *Shiraz E-Medical Journal, 23*. doi: 10.5812/semj-123479

9. Pierce LL, Steiner VL, Khuder SA, Govoni AL, Horn LJ. The effect of a Web-based stroke intervention on carers' well-being and survivors' use of healthcare services. Disabil Rehabil. 2009;31(20):1676-84. doi: 10.1080/09638280902751972. PMID: 19479528.
