# Supplementary material for: The Effectiveness of Nurse-Led Telecare Consultations Among Patients Who Have Experienced a Stroke: Systematic Review and Meta-Analysis
Source: J Med Internet Res. 2025 Nov 27;27:e74149. doi: 10.2196/74149 (PMC12699255; doi:10.2196/74149)
Supplement: Multimedia Appendix 4 [file jmir_v27i1e74149_app4.docx]

**Multimedia Appendix 4 GRADE evidence profile**

| **Outcome** | **No. of Participants (Studies)** | **Effect (95% CI) / Direction** | **Certainty of Evidence** | **Downgrading Rationale** |
| --- | --- | --- | --- | --- |
| **Systolic blood pressure (SBP)** | 1,537 (4 RCTs: Irewall 2015 ^3^, Ögren 2018 ^5^, Kerry 2013 ^6^, MacKenzie 2013 ^1^) | MD = -4.83 mmHg (-12.51 to 2.85), No significant effect | **Low** ⭑⭑◯◯ | Downgraded 1 level for risk of bias (some concerns in randomization and intervention adherence), 1 level for very high heterogeneity (I² > 90%). |
| **Diastolic blood pressure (DBP)** | 1,537 (4 RCTs: Irewall 2015 ^3^, Ögren 2018 ^5^, Kerry 2013 ^6^, MacKenzie 2013 ^1^) | MD = -6.41 mmHg (-13.76 to 0.93), No significant effect | **Low** ⭑⭑◯◯ | Downgraded 1 level for risk of bias, 1 level for very high heterogeneity (I² > 90%). |
| **Low-density lipoprotein cholesterol (LDL-C)** | 1,144 (2 RCTs: Irewall 2015 ^3^, Ögren 2018 ^5^) | MD = 0.01 (-0.08 to 0.09), No significant effect | **Low** ⭑⭑◯◯ | Downgraded 1 level for risk of bias, 1 level for high heterogeneity (I² > 90%). |
| **Psychological burden (depression, anxiety)** | 1179 (4 RCTs: Kirkness 2017 ^2^, Boter 2004 ^4^, Mou 2023 ^7^, Kerry 2013 ^6^) | Mixed results: 2 positive, 2 null | **Low** ⭑⭑◯◯ | Downgraded 1 level for inconsistency (direction and magnitude of effect varied), 1 level for imprecision (small sample sizes, wide CIs). |
| **Quality of life** | 917 (2 RCTs: Boter 2004 ^4^, Kerry 2013 ^6^) | Mixed: 1 domain-specific improvement, 1 null | **Low** ⭑⭑◯◯ | Downgraded 1 level for inconsistency, 1 level for imprecision (few studies, small N). |
| **Medication adherence** | 56 (1 RCT: MacKenzie 2013 ^1^) | No significant effect | **Very low** ⭑◯◯◯ | Downgraded 1 level for risk of bias, 1 level for imprecision (single small RCT), 1 level for indirectness (high baseline adherence). |
| **Healthcare service use** | 771 (3 RCTs: Boter 2004 ^4^, Pierce 2009 ^8^, Mou 2023 ^7^) | Reduced ED visits/hospital admissions in some studies, no effect in others | **Low** ⭑⭑◯◯ | Downgraded 1 level for inconsistency, 1 level for imprecision (variable definitions, mixed results). |
| **Stroke recurrence** | 437 (2 RCTs: Kerry 2013 ^6^, MacKenzie 2013 ^1^) | No significant effect | **Very low** ⭑◯◯◯ | Downgraded 1 level for risk of bias, 1 level for imprecision (few events), 1 level for indirectness (short follow-up, low baseline risk). |
| **Survivor functioning / coping** | 162 (1 RCT: Mou 2023 ^7^) | Significant improvement in coping at 3 months | **Low** ⭑⭑◯◯ | Downgraded 1 level for imprecision (single small study), 1 level for indirectness (short follow-up). |

**Notes:**

- CI = confidence interval; ED = emergency department; I² = heterogeneity statistic.
- Certainty ratings: **High** ⭑⭑⭑⭑, **Moderate** ⭑⭑⭑◯, **Low** ⭑⭑◯◯, **Very low** ⭑◯◯◯.
- Downgrading based on GRADE domains: risk of bias, inconsistency, indirectness, imprecision, publication bias.

Reference:

1. Mackenzie G, Ireland S, Moore S, Heinz I, Johnson R, Oczkowski W, Sahlas D. Tailored interventions to improve hypertension management after stroke or TIA--phase II (TIMS II). Can J Neurosci Nurs. 2013;35(1):27-34. PMID: 23687780.

2. Kirkness CJ, Cain KC, Becker KJ, Tirschwell DL, Buzaitis AM, Weisman PL, McKenzie S, Teri L, Kohen R, Veith RC, Mitchell PH. Randomized trial of telephone versus in-person delivery of a brief psychosocial intervention in post-stroke depression. BMC Res Notes. 2017 Oct 10;10(1):500. doi: 10.1186/s13104-017-2819-y. PMID: 29017589; PMCID: PMC5633890.

3. Irewall A-L, Ögren J, Bergström L, Laurell K, Söderström L, Mooe T (2015) Nurse-Led, Telephone-Based, Secondary Preventive Follow-Up after Stroke or Transient Ischemic Attack Improves Blood Pressure and LDL Cholesterol: Results from the First 12 Months of the Randomized, Controlled NAILED Stroke Risk Factor Trial. PLoS ONE 10(10): e0139997. https://doi.org/10.1371/journal.pone.0139997

4. Boter H; HESTIA Study Group. Multicenter randomized controlled trial of an outreach nursing support program for recently discharged stroke patients. Stroke. 2004 Dec;35(12):2867-72. doi: 10.1161/01.STR.0000147717.57531.e5. Epub 2004 Oct 28. PMID: 15514186.

5. Ögren J, Irewall AL, Söderström L, Mooe T. Long-term, telephone-based follow-up after stroke and TIA improves risk factors: 36-month results from the randomized controlled NAILED stroke risk factor trial. BMC Neurol. 2018 Sep 21;18(1):153. doi: 10.1186/s12883-018-1158-5. PMID: 30241499; PMCID: PMC6148791.

6. Kerry SM, Markus HS, Khong TK, Cloud GC, Tulloch J, Coster D, Ibison J, Oakeshott P. Home blood pressure monitoring with nurse-led telephone support among patients with hypertension and a history of stroke: a community-based randomized controlled trial. CMAJ. 2013 Jan 8;185(1):23-31. doi: 10.1503/cmaj.120832. Epub 2012 Nov 5. PMID: 23128283; PMCID: PMC3537777.

7. Mou H, Lam SKK, Chien WT. The effects of a family-focused dyadic psychoeducational intervention for stroke survivors and their family caregivers: A randomised controlled trial. Int J Nurs Stud. 2023 Jul;143:104504. doi: 10.1016/j.ijnurstu.2023.104504. Epub 2023 Apr 17. PMID: 37149953.

8. Pierce LL, Steiner VL, Khuder SA, Govoni AL, Horn LJ. The effect of a Web-based stroke intervention on carers' well-being and survivors' use of healthcare services. Disabil Rehabil. 2009;31(20):1676-84. doi: 10.1080/09638280902751972. PMID: 19479528.
